# Development and validation of the felt safeness in the therapeutic relationship questionnaire

**DOI:** 10.1111/papt.70078

**Published:** 2026-05-13

**Authors:** Emily Hesselink, Jasmin Langdon‐Daly

**Affiliations:** ^1^ Department of Psychology, University of Bath Bath UK

**Keywords:** psychometric properties, social safeness, therapeutic relationship, therapy outcome, validation

## Abstract

**Objective:**

To develop and evaluate the Felt Safeness in the Therapeutic Relationship Questionnaire (FSTRQ), a novel measure of someone's sense of social safeness within the therapeutic relationship.

**Method:**

226 participants completed online questionnaires including FSTRQ and measures of overall social safeness, therapeutic alliance, positive/negative affect and worry. Confirmatory factor analysis (CFA) examined the fit of the study data to a hypothesised one‐factor structure. Reliability statistics were calculated, and correlational analyses were used to explore convergent and discriminant validity.

**Results:**

The CFA showed an adequate model fit of the one‐factor structure to the data from the FSTRQ. Cronbach's alpha for the measure was 0.96. Convergent and divergent validity were supported by strong correlations with the questionnaire related to therapeutic alliance, moderate correlations to the SSPS and positive affect subscale and no correlations with negative affect and worry.

**Conclusion:**

This study showed good validity and reliability of the FSTRQ as a unique measure of felt safeness in the therapeutic relationship.

## INTRODUCTION

Social safeness is an affective, emotional state characterised by feelings of being safe, connected, warm and cared for within one's social world. It represents the action of a soothing, affiliative system that reduces anxiety, fosters trust and is often linked to early experiences of care and the attachment concept of ‘secure base’ (Gilbert, 2024). Models of therapeutic mechanisms such as the contextual model frame change in psychological therapies as arising partly through relational experience with the therapist acting directly via evolved socio‐emotional mechanisms (the so‐called ‘real relationship’) (Wampold, 2015). An experience of safeness has been suggested as a one such mechanism by which the relationship with the therapist creates and supports change, supporting trust and openness, courage and exploration, and the regulation of threat and shame (Gilbert, 2024). As yet, no measure exists to support measurement or empirical investigation of the impact of felt safeness in the therapeutic relationship.

### Social safeness

Social safeness is a paradigm used to describe the positive feeling that is generated when experiencing affiliative actions from others (Gilbert et al. 2008; Gilbert, 2024). Examples of affiliative behaviour include facial expressions, physical touch and access to care (Gilbert & Leahy, 2007). Depue and Morrone‐Strupinsky (2005) outline that there are two types of positive affect: the safe/content form of positive affect which reflects happiness, contentment and relief; as opposed to high‐arousal positive affect such as excitement or desire. Gilbert (2024) describes experiences of social safeness leading to safe/content positive affect through the activation of the affiliative/soothing system component of the tripartite model of emotion regulation. This model of emotion incorporates three interacting emotional systems: the drive system (arousal state leading to pursuit of reward and forms of positive affect such as excitement), the threat system (responsive to a range of present or imagined threats, including threats to survival, social rejection, exclusion and conflict, worry and stress, and involving activation of the sympathetic nervous system), and the affiliative/soothing system (generating feelings of calmness, contentment, approach to others, and safeness to explore through affiliative behaviours) (Gilbert, 2024).

The affiliative/soothing system is described as an evolved system which emerges and is activated in contexts of attachment relationships and interpersonal care and support. It involves dopamine, endorphin and oxytocin action to motivate care and affiliative actions (Gilbert, 2024). Safeness is the cognitive‐emotional experience of the activity of this system. More than just the perceived absence of threat (‘safety’), safeness involves a perception of others as available to help, trustworthy and caring, along with the emotional experience of felt ‘safeness’. This experience facilitates the potential for relationships (with others who are physically present, or present in the mind or memory) to act as a ‘secure base’ or ‘safe haven’, supporting approach and trust in others, play, learning, exploration and the formation of mutually beneficial social connections, while also acting to inhibit threat and drive systems (Gilbert, 2009, 2024). The action of the affiliative/soothing system and the experience of safeness therefore help individuals cope with challenges that occur within their life, while a less developed or activated soothing system and reduced feelings of social safeness can be maladaptive. Reduced experience of social safeness is common across many different types of psychopathology including disordered eating and depression (Alavi, 2021; Kelly et al., 2012; Langdon‐Daly et al., 2024). Conversely, repeated experiences of care, and activation of the soothing system could reduce feelings of threat and improve ability to process adverse life events (Gilbert & Leahy, 2007). For example, good parent–child relationships can have a protective influence over psychopathology (Mathijssen et al., 1998).

### Social safeness in the therapeutic relationship

Activating the affiliative/soothing system and creating safeness within the therapeutic relationship may be beneficial in psychotherapy (Gilbert, 2024). Change in psychological therapies occurs in the context of the relationship and interactions between the therapist and the client (Norcross & Wampold, 2011; Wampold, 2015). It often involves discussions about highly emotive and sensitive topics, and anxiety‐provoking behavioural change. A good therapeutic relationship may directly support or create positive change in part by generating increased feelings of social safeness within therapy sessions, and a perception of the therapist as trustworthy, compassionate and motivated to help, allowing clients to share their experiences more openly, experience courage in facing difficulties and making changes, and regulating threat activation and shame (Gilbert & Leahy, 2007). Although the association and role of therapeutic relationship with therapy outcome is widely evidenced and accepted (e.g., Falkenström et al., 2013) at present, the role of safeness in the therapeutic relationship as a potential mechanism for change has been theorised but not empirically established, since there is currently no measure of social safeness within the therapeutic relationship. Assessing social safeness within the specific relationship between a client and therapist is important, as it facilitates research which could highlight a useful therapeutic target which influences therapy outcome, and which could potentially be modifiable through shifts in therapist behaviour or approach.

### Existing measures for related constructs

Social safeness is predominantly measured using the Social Safeness and Pleasure Scale (SSPS) which is focussed on the positive affect felt in relationships ‘in general’ (Gilbert, 2009). Statements describe an individual's tendency to experience social safeness and safe/content positive affect in relationships. The original study showed a one‐factor structure for the questionnaire, which has since been replicated, suggesting that there is one latent factor, underlying all of the items in the questionnaire, which is hypothesised to reflect feelings of social safeness (Alavi, 2021; Gilbert, 2009).

The most commonly described and measured facet of the therapeutic relationship is therapeutic alliance or ‘working alliance’, described as the therapist and clients mutual engagement in the work of therapy (Stubbe, 2018). Commonly used measures such as the Vanderbilt Therapeutic Alliance Scale (VTAS) and the Working Alliance Inventory (WAI) are based on Bordin 1979's three‐part model of therapeutic alliance, which describes a working relationship between therapist and client that requires agreement on shared goals and tasks of therapy, along with the development of an affective personal bond (Hartley & Strupp, 1983; Horvath & Greenberg, 1989). Therapeutic alliance has been consistently demonstrated as predictive of the outcome of psychotherapeutic interventions, with a stronger alliance associated with improved outcomes (Horvath & Symonds, 1991; Martin et al., 2000). Whilst these measures are well established and useful in clinical settings, they do not assess the experience of ‘safeness’ specifically, as another potentially relevant facet of the therapeutic relationship.

### Aims and objectives

The Felt Safeness in the Therapeutic Relationship Questionnaire (FSTRQ) is an 11‐item measure focussed on felt safeness specifically in the therapeutic relationship. The measure is an adaptation of the original SSPS (Gilbert, 2009), with items adapted to shift the focus specifically towards how a client feels about their therapist. This study aims to:

- Assess the reliability and validity of the FSTRQ measure in a group of participants who have experienced any form of psychotherapy within the last 3 years.
- Consider whether felt safeness can be measured in relation to a single relationship rather than in relationships in general.
- To explore associations of safeness in the therapeutic relationship with general social safeness, therapeutic alliance, affect and worry.

## METHOD

### Participants

Participants were 226 English speaking individuals over the age of 16 who self‐reported having experienced any type of psychological/psychotherapy within the past 3 years, with at least three therapy sessions with the same therapist. One hundred eight of these completed the second instance of the FSTRQ (for calculation of test re‐test reliability). Participants were predominantly female (58.9%) with a modal age range of 25–34 (41.6%). Demographic characteristics for the two datasets (Prolific and general recruitment) and the combined data set are shown in Table 1.

**TABLE 1:** Demographic characteristics of participants.

Demographics	General		Prolific		Full data set	
	*n*	%	*n*	%	*n*	%
**Age range**						
16–24	34	35.1	22	17.1	56	24.8
25–34	40	41.2	54	41.9	94	41.6
35–44	11	11.3	27	20.9	38	16.8
45–54	7	7.2	15	11.6	22	9.7
55–64	2	2.1	8	6.2	10	4.4
65–74	3	3.1	2	1.6	5	2.2
75–84	0	n/a	1	0.8	1	0.4
**Gender**						
Female	70	72.2	63	48.8	133	58.9
Male	25	25.8	59	45.7	84	37.2
Transgender	0	n/a	1	0.8	1	0.4
Non‐binary	0	n/a	4	3.1	4	1.8
Prefer not to say	1	1.0	1	0.8	1	0.4
Multiple identities	1	1.0	2	1.6	3	1.3
**Ethnicity**						
Asian/Asian British	8	8.24	3	2.3	11	4.9
Black/Black British/Caribbean/African	13	13.4	39	30.2	52	23.0
Mixed or multiple ethnic groups	4	4.12	3	2.3	7	3.1
White	67	69.1	77	59.7	144	63.7
Prefer not to say	1	1.0	2	1.6	3	1.3
Other	4	4.1	5	3.9	9	4.0
**Level of education**						
No formal qualifications	1	1.0	5	3.9	6	2.7
GCSEs or equivalent	6	6.2	2	1.6	8	3.5
A Levels or equivalent	11	11.3	11	8.5	22	9.7
Apprenticeship	0	n/a	0	n/a	0	n/a
Undergraduate degree	46	47.4	61	47.3	107	47.3
Master's degree	26	26.8	29	22.5	55	24.3
Professional qualifications	3	3.1	3	2.3	6	2.7
Doctorate	3	3.1	15	11.6	18	8.0
Prefer not to say	1	1.0	2	1.6	3	1.3
Other	0	n/a	1	0.8	1	0.4
**Total *n* values**						
*n*	97	42.9^a^	129	57.1^a^	226	n/a

*Note*: The frequencies and percentages shown for the two categories of data collection and the full data set used.

^a^ The percentage calculated is based on the *n* value for the full data set.

Participants were recruited via social media, internal University recruitment systems, websites targeted at recruiting research participants and Prolific, a paid research participant platform (www.prolific.com, 2025). Study advertisements invited participants to take part in a study to test a questionnaire which asks about how emotionally safe and supported you felt with your therapist. The decision to recruit via Prolific as well as unpaid recruitment avenues was made part way through data collection, to support timely recruitment of adequate participant numbers. Prolific was chosen as it has been demonstrated as providing high quality data in terms of attention, comprehension, honesty and reliability (Peer et al., 2022).

The inclusion criteria of therapy within the last 3 years was chosen to balance maximising the potential participant pool to support adequate recruitment, with the need for participants to be able to recall their therapy experience in sufficient detail. Checks were made in order to protect against fraudulent research participation, with six participants from the Prolific group and eight participants from the general recruitment group identified as potentially fraudulent and excluded. Exclusion of potentially fraudulent research participants was undertaken in line with guidance detailed by Mayer et al., 2025, in advance of data analysis, as detailed in the pre‐registration for this study (see Figure S1). These included:

- A participant screening questionnaire for Prolific‐based participants, with participants not aware of full inclusion criteria in advance (i.e. length of time since therapy).
- Manual screening of email address/ response time/ IP address to identify potential ‘spam’/‘bot’ participants. Email addresses were checked visually to ensure they had a valid format, with IP addresses only checked following a reason for concern. No participants were excluded based on response time or time stamps for the onset of each questionnaire being too close together.
- Asking participants to briefly indicate the presenting issue they received therapy for and the kind of therapy. Responses were reviewed manually, with participants giving non‐sensical or clearly inconsistent responses (eg presenting issue given as ‘Limited Applicability’) excluded.

### Measures

#### Felt Safeness in Therapeutic Relationship Questionnaire (FSTRQ)

A novel, 11‐item questionnaire adapted by the research supervisor (JLD, a clinical psychologist) from the SSPS developed by Gilbert (2009). The FSTRQ was submitted to the Compassionate Mind Foundation and approval was given for adaptation of the SPSS and use of the adapted measure in this project (Compassionate Mind Foundation, personal communication, 12th November 2024). The language of all items in the scale was changed to reflect the focus on the therapeutic relationship, referring exclusively to ‘*my therapist*’. A number of further changes were made following feedback from piloting and review by a Speech and Language Therapist, and review of best practice guidance on measure design. One of the items, ‘*I feel part of something greater than myself’*, from the original SSPS was removed, as it could not be easily adapted to a focus on a specific relationship. An item about feeling ‘secure and wanted’ was separated into two items (‘secure with’ and ‘valued by’) to ensure each item included only one construct. Written descriptor labels were added for each response option (Artino & Gehlbach, 2012; Swain et al., 2008).

The questionnaire involves rating 11 items such as ‘*I feel valued by my therapist’* on a five‐point Likert scale where 1 is ‘*Almost never’* and 5 is ‘*Almost all the time’*. Higher summed scores indicate a client experience of stronger social safeness with the therapist.

#### Social Safeness and Pleasure Scale (SSPS)

An 11‐item, self‐report questionnaire that assesses the level of social safeness someone feels in their life and relationships in general. Individual items such as ‘*I find it easy to be calmed by those around me’* are rated on a five‐point Likert Scale ranging from 0 to 4, with higher summed scores indicating increased security and feelings of safeness in relationships with others. The initial scale was found to be highly reliable with a Cronbach's alpha of 0.91, and subsequent studies have replicated the high reliability in other languages and clinical groups (Alavi, 2021; Gilbert, 2009; Isaksson et al., 2022).

#### Working alliance inventory short form (WAI‐SR)

A 12‐item scale assessing the therapeutic alliance between client and therapist, based on the three‐part model of working alliance (personal bond, agreement on goals and agreement on tasks of therapy). Statements such as ‘*What I am doing in therapy gives me new ways of looking at my problem’* are rated on a five‐point Likert Scale with scores ranging from 1 to 5 (Horvath, 1994). The higher the summed score, the greater the strength of the therapeutic alliance. The WAI‐SR shows a high reliability with Cronbach's alpha lying between .91 and .92 (Hatcher & Gillaspy, 2006).

#### Positive and negative affect schedule (PANAS)

A 20‐item scale measuring 10 items of positive affect and 10 of negative affect was used to assess general current affect. Participants indicate how often they have felt mood states such as ‘*Interested’* over the past week on a five‐point Likert Scale ranging from 1 ‘*Very slightly or Not at all’* to 5 ‘*Extremely’*, Items are split into two groups, positive affect (PA) items (PANAS‐PA) and negative affect (NA) items (PANAS‐NA), which are summed separately with higher values indicating greater positive and negative affect, respectively. Cronbach's alpha values range from 0.86 to 0.90 for PA and 0.84 to 0.87 for NA, with a test–retest reliability of .81 for PA and .79 for NA (Watson et al., 1988).

#### Penn state worry questionnaire—abbreviated (PSWQ‐A)

An eight‐item scale assessing how strongly an individual tends to experience worry. Items such as ‘*When I am under pressure, I worry a lot’* are rated on a five‐point scale and summed to give an overall score, where higher values indicate increased levels of worry (Meyer et al., 1990). The abbreviated version has good reliability and validity with a Cronbach's alpha of .87 and has a one‐factor structure indicating assessment of one latent factor (Hopko et al., 2003). The abbreviated version was used due to the improved model fit and more time‐efficient quality compared to the original measure (Hopko et al., 2003).

### Procedure

This study was pre‐registered to the Open Science Framework. Study data was collected and managed by the online platform REDCap at the University of Bath (Harris et al., 2009, 2019). Participants viewing study advertisements followed a link to a Participant Information and Consent page which provided comprehensive study information and asked participants to indicate consent to participate in the study. Participants were then asked preliminary questions on the type of therapy that they had, main presenting issue, and the format of their therapy (ie online/in person). Participants were then asked to complete five different scales: the FSTRQ, the SSPS, the WAI‐SR, the PANAS and PSWQ‐A. The SSPS, WAI‐SR and PSWQ‐A contained attention questions to confirm the participant was concentrating on the task (e.g., Attention check: Please select ‘Fairly often’). At the end of the first part of the study, participants were asked for demographic information including their age, gender, ethnicity and education level, before being directed to a debriefing page containing information about mental health support plus a link or completion code for reimbursement (for Prolific participants) or space to enter email to enter a prize draw for one of four £20 Amazon vouchers (for participants from other recruitment routes).

After completing the first part of the study, all participants were invited to complete a second instance of the FSTRQ questionnaire to determine the test–retest reliability. Email invitations were sent 7 days after completion of the first part of the study. The second instance of the study featured only a consent page, the FSTRQ and a second debrief page. A test–retest interval of 1 week was chosen to balance study retention with memory effects. This interval is considered appropriate for measures of constructs which are expected to remain relatively stable, such as describing a therapeutic relationship which occurred in the past and has ended (Polit, 2014).

### Statistical analysis

Data were visually inspected, reorganised and summed using Microsoft Excel. Incomplete entries, where more than one of the responses to each individual questionnaire were left blank by a participant, were removed (*n* = 24), along with data for suspected fraudulent entries (*n* = 14) and data for participants answering more than one attention question incorrectly (*n* = 6), as detailed above. All statistical analyses were completed using R software version 4.5.1 and included packages *lavaan*, *ppcor blandr*, *corrplot, ggplot2, EFAtools* and *dplyr* (Datta, 2017; Kim, 2015, R Core Team, 2025; Rosseel, 2012; Steiner & Grieder, 2020; Wei & Simko, n.d. (24 C.E.); Wickham, 2016; Wickham et al., 2014). Data were analysed to check for violations to assumptions of normality and for any skew within the data. The two data sets (participants gained through Prolific and participants gained through other recruitment) were compared using ANOVA to assess for significant differences in demographics and questionnaire responses between the two data sets.

A confirmatory factor analysis (CFA) was completed to confirm a one factor structure for the FSTRQ. As our questionnaire is adapted from the original SSPS, we hypothesised that it would reflect the one factor structure shown in the original measure (Alavi et al., 2017; Gilbert, 2009). WLSMV analysis was used instead of maximum likelihood analysis, as this is considered more robust to small samples with non‐parametric, ordinal data (Finney & DiStefano, 2013). Factor loadings should be greater than 0.7 for reliable interpretation of the data (Hair et al., 2009). A range of indices were used to provide a more comprehensive assessment of model fit. Calculating chi squared used maximum likelihood methods to assess model fit (Brown, 2015). Absolute model fit measures given included the root mean square error of approximation (RMSEA) and the associated 90% confidence intervals as well as the standardised root mean square residual (SRMR) (Brown, 2015). Relative model fit was assessed using the Comparative Fit Index (CFI) and the Tucker–Lewis Index (TLI) (Bentler & Bonett, 1980; Tucker & Lewis, 1973). Hu and Bentler (1999) suggested that acceptable CFI and TLI values should be greater than 0.95 and SRMR values should be smaller than 0.08. RMSEA < 0.05 shows a close fit of the model; RMSEA between 0.05 and 0.08 are a reasonably close fit; 0.08–0.10 shows marginal fit; and RMSEA >0.10 is considered an unacceptable model fit (Fabrigar et al., 1999). Chi squared (χ^2^) values should be statistically non‐significant for an ideal fit and the ratio df: χ^2^ ≤ 2 (Alavi et al., 2020; Brown, 2015).

Internal consistency was assessed using Cronbach's alpha with a value of >0.70 being considered acceptable (Cronbach, 1951; Taber, 2018). We analysed test–retest reliability using Bland–Altman's Limits of Agreement analysis to generate graphs of the data based on upper and lower limits of the differences between pairs of data (Bland & Altman, 1986). If the test–retest reliability is sufficient, 95% of the data should lie between the upper and lower limits of agreement (Gerke, 2020). This analysis was completed in conjunction with a correlation analysis in order to give a more comprehensive assessment of test–retest reliability.

Convergent and divergent validity were assessed using Spearman's correlation analysis as the data is ordinal. Group membership was controlled for using partial correlation due to significant differences in age and responses to the WAI‐SR, PANAS‐PA, PANAS‐NA and PSWQ‐A across groups (van Aert, 2023). A strong correlation between the FSTRQ, the SSPS and WAI‐SR, a medium sized correlation with the PANAS‐PA (since safeness is defined in terms of positive emotional experience), and small negative or no significant correlations with the PSWQ‐A and the PANAS‐NA would be expected to confirm convergent and divergent validity. *r* values closer to 1 indicate a stronger relationship between two variables (Schober et al., 2018). This pattern of associations with the PANAS was expected since the FSTRQ refers to a past relationship while the PANAS refers to current affect, reflecting that safeness is defined as a distinct form of positive emotional experience, and distinct from the absence of negative emotion/worry (Armstrong et al., 2021; Depue & Morrone‐Strupinsky, 2005; Gilbert, 2024).

### Ethical approval

Ethical approval for this study was gained from the University of Bath Social Sciences Research Ethics Committee. Reference: 8536.

## RESULTS

### Preliminary analyses

There was no significant difference between the two data sets (Prolific vs. general recruitment) in response to the FSTRQ (*F*_(1,224)_ = 1.26, *p* = .26). The Prolific dataset had a significantly higher modal age than the general recruitment dataset (*F*_(1,224)_ = 7.99, *p* < .05), however, this value still lies within the same age range (25–34). There were significant differences between groups in scores on the WAI (higher in Prolific group), PANAS‐positive affect (higher in Prolific group) and PSWQ worry (lower in Prolific group). None of the other demographic variables or measures showed significant differences (see Table 2 below).

**TABLE 2:** ANOVA analyses comparing Prolific and general recruitment data sets.

Item	General (*n* = 97)		Prolific (*n* = 129)		*F*_(1,224)_	*η*_*p*_^*2*^	Demographic label (modal)
	Mean	SD	Mean	SD			
**Questionnaire**							
FSTRQ	44.1	9.55	45.42	8.02	1.26	< 0.01	
WAI‐SR	46.08	10.97	51.15	9.07	14.41*	0.06	
SSPS	36.94	9.27	39.05	9.08	2.96	0.01	
PANAS‐PA	29.33	8.64	33.12	9.88	9.00*	0.04	
PANAS‐NA	24.56	7.29	20.26	7.42	18.82*	0.08	
PSWQ‐A	28.82	6.62	25.92	7.73	8.80*	0.04	
**Demographics**							
Age	2.09	1.20	2.56	1.24	7.99*	0.03	25–34
Gender	1.53	2.28	1.91	3.09	1.09	< 0.01	Female
Ethnicity	3.54	1.14	3.4	1.12	0.86	< 0.01	White
Education	5.01	1.43	5.35	1.68	2.56	0.01	Undergraduate degree

* *p* < .*05*.

Visual inspection of Q‐Q plots and histograms of the data showed that the FSTRQ responses were skewed to the left (skewness = −1.23, kurtosis = 4.83) with a Shapiro–Wilk test indicating that the data was not normally distributed (W = 0.89, *p* < .05).

### Factor structure

The factor structure was dictated as a one‐factor model where all of the items mapped onto a single latent variable (shown in Figure 1). Six entries containing missing values were excluded from this analysis.

**FIGURE 1:**
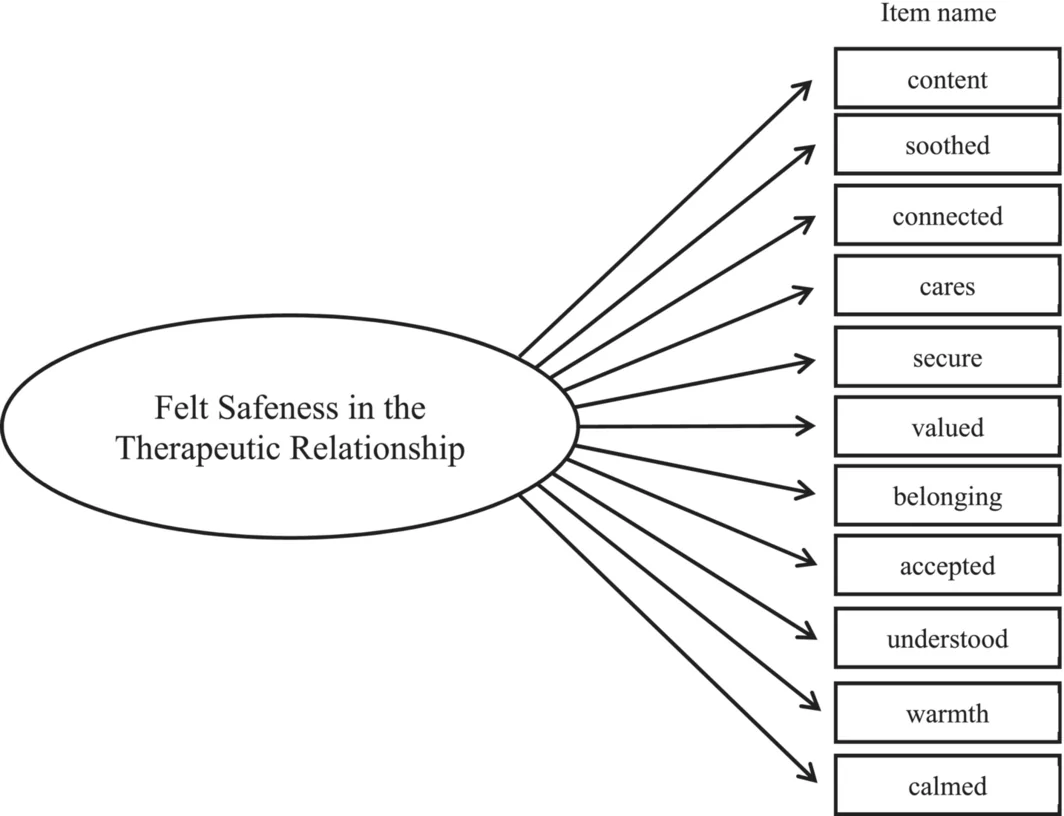
Factor structure of the FSTRQ. The single latent factor mapping onto all of the items of the FSTRQ.

Fit indices suggested a marginal fit between the model and the data (Chi squared (df = 44, *n* = 220) = 114.55, *p* < .05, RMSEA = 0.086 (90% CI [0.067, −0.105]), SRMR = 0.033, CFI = 0.99, TLI = 0.99). The factor loadings all ranged between 0.84 and 0.91 and are summarised in Table 3. Overall, the data showed a marginal fit to the one‐factor model.

**TABLE 3:** Factor loadings from confirmatory factor analysis.

Item no./variable name	Item wording	Factor loading
1 – content	I feel content with my therapist	.86
2 – soothed	I feel easily soothed by my therapist	.85
3 – connected	I feel connected to my therapist	.88
4 – cares	I feel that my therapist cares about me	.84
5 – secure	I feel secure in my relationship with my therapist	.87
6 – valued	I feel valued with my therapist	.90
7 – belonging	I feel a sense of belonging when I am with my therapist	.85
8 – accepted	I feel accepted by my therapist	.86
9 – understood	I feel understood by my therapist	.86
10 – warmth	I feel a sense of warmth in my relationship with my therapist	.91
11 – calmed	I find it easy to feel calmed by my therapist	.91

*Note*: Factor loadings represent the extent to which the item contributes to the outcome of the FSTRQ. All of the values are above the satisfactory threshold of 0.70. The items and variable names from the questionnaire are included for reference.

### Reliability

Cronbach's alpha was 0.96 for the sample, showing good internal consistency. For test–retest reliability, there was a strong, positive correlation between the two instances of the FSTRQ (r = 0.86, 95%, 95% CI [0.81, 0.90]; *p* < .05). Agreement between the two instances of the FSTRQ (*n* = 128) was further determined by Bland Altman Limits of Agreement. The mean difference between the two responses to the FSTRQ was 2.66. The limits were determined as −7.85 (95% CI [−9.23, −6.46]) and 10.24 (95% CI [8.85, 11.62]). Most of the data points fell within the two limits of agreement (see Figure 2).

**FIGURE 2:**
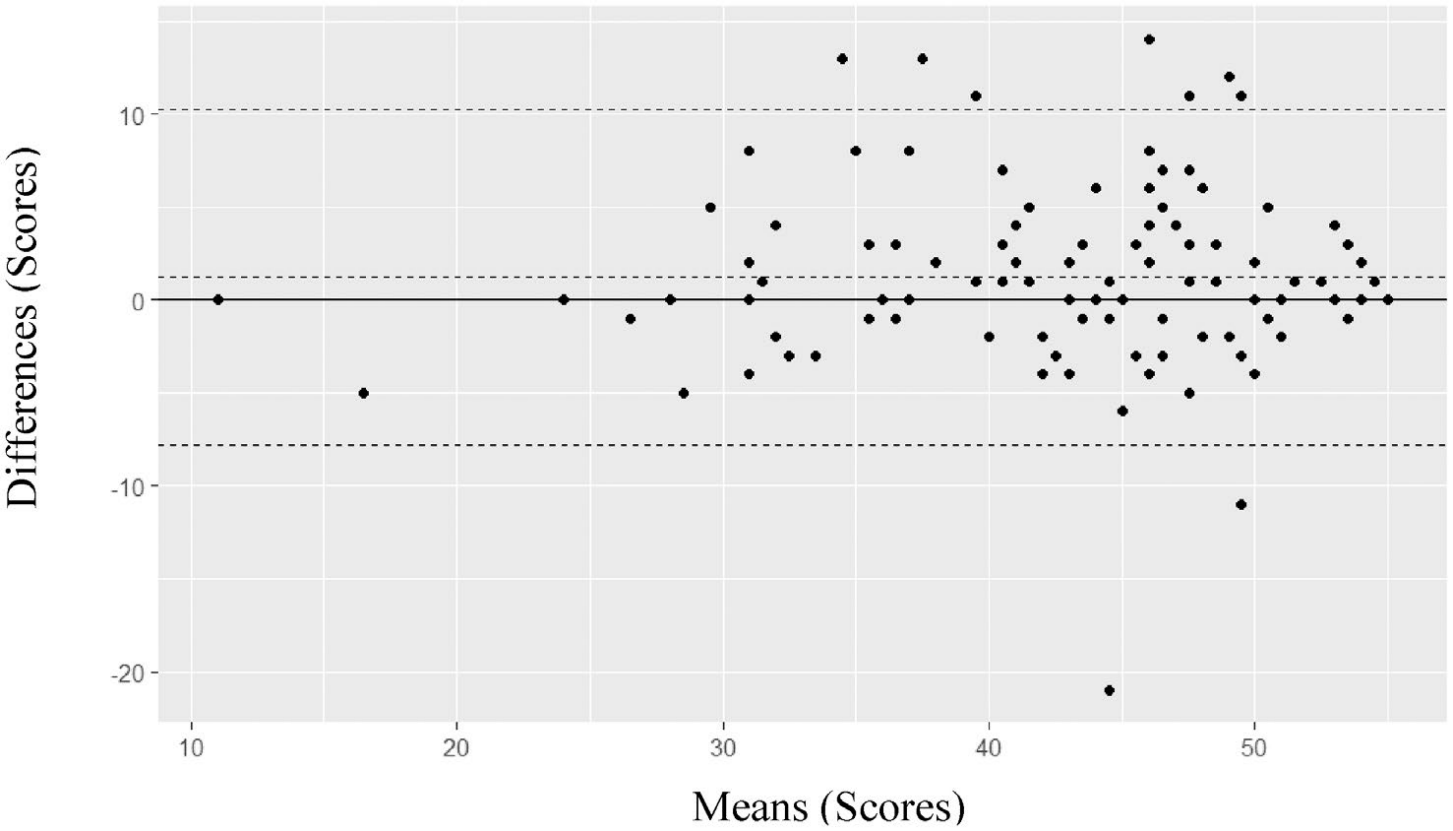
Agreement between Time 1 and Time 2 of FSTRQ using Bland–Altman limits of agreement. Dashed lines show the Bland‐Altman Limits of Agreement.

### Convergent and discriminant validity

Safeness in the therapeutic relationship (FSTRQ) showed a strong, positive correlation with therapeutic alliance (WAI‐SR) (r = .82, *p* < .05), and a medium‐sized positive correlation with general social safeness (SSPS) (r = .4, *p* < .05) and positive affect (PANAS‐PA) (r = .43, *p* < .05) showing strong convergent validity. There was no correlation with worry (PSWQ‐A) (r = −.08, *p* < .32) or negative affect (PANAS‐NA) (r = −.07, *p* = .25) showing divergent validity. The correlations are shown in the correlation matrix (Table 4). The pattern of correlations was consistent with expected convergence/divergence to theoretically related or unrelated constructs.

**TABLE 4:** Correlation matrix for all of the questionnaires in Part 1 of the study.

	FSTRQ	WAI‐SR	SSPS	PANAS (PA)	PANAS (NA)	PSWQ‐A
FSTRQ	‐					
WAI‐SR	.82*	‐				
SSPS	.44*	.47*	‐			
PANAS (PA)	.42*	.47*	.67*	‐		
PANAS (NA)	−.07	−.08	−.34*	−.19*	‐	
PSWQ‐A	−.08	−.10	−.30*	−.28*	.44*	‐

Abbreviations: FSTRQ = Felt Safeness in Therapeutic Relationship Questionnaire, PANAS PA/NA = Positive and Negative Affect Scale Positive Affect/Negative Affect, SWQA = Penn‐State Worry Questionnaire Abbreviated SSPS = Social Safeness and Pleasure Scale, WAI‐SR = Working Alliance Inventory‐Short Revised.

* *p* < .05. Partial correlations used to control for group membership.

## DISCUSSION

Social safeness is an emotional experience suggesting activation of the soothing system in our relationships with others (Gilbert, 2024). Our measure was developed to assess felt safeness within one particular relationship: between a client and their therapist. It has been suggested that an experience of social safeness in the therapeutic relationship might be relevant to, or a mechanism of change in, psychotherapy, and potentially predictive of therapy outcome (Gilbert & Leahy, 2007; Gilbert, 2024). In this study, we aimed to assess the psychometric properties of the FSTRQ measure and confirm whether this tool reliably assessed felt safeness within the therapeutic relationship. Acceptable model fit was observed from multiple measures of fit for a one‐factor structure for the FSTRQ, which matched the factor structure observed in the SSPS (Gilbert, 2009; Isaksson et al., 2022). The Chi squared value indicates marginal fit of the model, however, this measure of fit is heavily influenced by sample size and may reflect the smaller size of our sample (Alavi et al., 2020). Reliability measures showed excellent internal consistency (Cronbach's alpha = .96) and reasonable test–retest reliability. This figure is similar to the alpha values found in validation studies of the original SSPS scale which our measure was adapted from (Gilbert, 2009; Isaksson et al., 2022). While some texts suggest that alpha should be maximised, other suggest that excessively high alpha values (e.g., higher than around 0.7), and associated high inter‐item correlations, may indicate item redundancy (Kline, 1979; Ray, 1988). In this study, mean inter‐item correlation was 0.68, with some individual correlations higher than 0.7, but no individual items consistently more highly inter‐correlated than others. The decision to remove ‘redundant’ items requires consideration of both statistical metrics and qualitative consideration of separateness of construct assessed. New algorithmic methods have also been suggested to create abbreviated measures, such as use of a Genetic Algorithm (see Passarelli et al., 2024). As an adaptation of an existing measure, we have not identified specific items for removal in this study.

Convergent and divergent validity were indicated by an as predicted pattern of correlations with theoretically similar and distinct constructs. There were moderate to strong correlations between the FSTRQ and the working alliance, social safeness, and positive affect measures, and no correlations with the worry and negative affect measures, respectively.

The results showed that the SSPS could be successfully adapted for a single relationship. The FSTRQ was more highly correlated with the working alliance measure than the SSPS, suggesting that it is measuring some characteristic of the same specific relationship (between the client and therapist), rather than a general tendency. The finding that feelings of safeness felt in the therapeutic relationship are only moderately associated with safeness in relationships general suggests potential for the therapeutic relationship to be a different or new relational and emotional experience, including for clients living in more hostile social environments or with difficult attachment histories. This finding is in line with theoretical accounts of safeness as a potential mechanism and driver of therapeutic change (Gilbert, 2024). The relevance of safeness to wellbeing and mood is demonstrated by the findings of a positive association of safeness (generally, and in the therapeutic relationship) with positive affect, along with weaker negative associations (of general safeness) with negative affect and worry. The suggestion that experience of felt safeness in the therapeutic relationship might generalise to shifts in general safeness or affect would need to be considered in studies assessing these variables before, during and after therapy experiences. The psychometric properties of the FSTRQ suggest that the SSPS could also be adapted to refer to and assess other single relationships such as parent–child or romantic relationships, while still measuring felt safeness.

The very high correlation between this measure and the therapeutic alliance suggests careful consideration is required of whether the construct of felt safeness in the therapeutic relationship is meaningfully distinct from the broader concept of therapeutic alliance. We might expect them to be closely linked, and there is potential in this retrospective study that generalisation of recall of different aspects of a past relationship to be more globally positive or negative has occured, reducing discrimination of subtly different aspects of the experience (Anvari et al., 2024). Future studies should assess these variables ‘live’ in the process of active therapy, and critically consider whether measuring the construct of safeness adds more explanatory value to models of therapeutic outcomes than therapeutic alliance alone.

## LIMITATIONS

This study has several limitations which are important to address. Firstly, this study uses solely self‐report measures over an online platform. The use of self‐report measures is appropriate as a means to access the clients' perceived emotional experience within the therapeutic relationship (Horvath, 2000). However, as with all self‐report measures there is a risk of skew related to response tendencies. As feeling safe within the therapeutic relationship and feelings of positive affect could be seen as socially desirable, participants may have responded more positively to the FSTRQ than they felt. Participants were also asked to report on a relationship occurring up to several years in the past. As acknowledged above, this may have impacted on response tendency. Participants generally responded well to attention questions, suggesting that the FSTRQ and other related questionnaires were an adequate length to hold the attention of the participant. Attention questions were included in three of the questionnaires to try and assess levels of concentration throughout the study. All of the existing measures had strong psychometric properties, suggesting they are appropriately reliable self‐report measures.

Secondly, the FSTRQ was developed solely by the research team, without co‐production with patients or other clinicians, and no qualitative feedback was requested on the measure. As far as this author is aware, the SSPS which this scale was adapted from has also not been developed from or received qualitative public/patient feedback. Contemporary guidance on the evaluation of new patient reported outcome measures highlights the value of considering ‘content validity’ (defined as relevance, comprehensiveness and comprehensibility of items), which is promoted through co‐production of measures with patients and clinicians and review and integration of qualitative feedback (Mokkink et al., 2019). This means that the questions included may not accurately represent the understandings and meanings of the target group—in this case individuals with recent experience of therapy. To improve this, future development or review of the measure should use focus groups and/or include some qualitative questions within the study design for participants to give their feedback on the FSTRQ.

Thirdly, non‐normal data were analysed using parametric statistical methods. Obtaining non‐normal data or ordinal data is common across validation studies for measures assessing psychological phenomena (Ferr, 2025; Spitzer et al., 2006). In this case, it would be likely for the data to have a left skew as more individuals should have good experiences with their therapists than bad experiences, given that therapists are highly trained individuals who would aim to foster a good therapeutic relationship (Bolsinger et al., 2020; Bourgon & Guiterrez, 2013; Smith, 2017). However, there may still be a ceiling effect in the data. This could be improved by increasing the sensitivity of the upper part of the scale, for example by increasing the number of points on the Likert scale to 7. There is some evidence that a Likert scale of 6 or 7 items could improve the applicability of the measure, particularly in groups with higher levels of education as was seen in our data set (Oaster, 1989; Weng, 2004).

Finally, there are limits to generalisability based on the population sample, with most participants belonging to the white ethnicity group and having reached at least a bachelor's degree level in education (de Oliveira & Baggs, 2023). Further research would need to assess the applicability of the FSTRQ to other ethnic groups and in other languages.

## FUTURE DIRECTIONS AND CLINICAL IMPLICATIONS

This study has furthered our knowledge of the role of social safeness in the therapeutic relationship and has shown that felt safeness can be measured in a single relationship. Gilbert and Leahy (2007) suggested that generating felt safeness may provide a mechanism for how the therapeutic relationship modulates therapy outcome. This occurs when increased felt safeness with the therapist causes activation of the soothing system, regulating threat/anxiety, allowing clients to feel comfortable in becoming more vulnerable and sharing more with their therapist, and supporting courage to make behavioural change (Gilbert, 2024; Gilbert & Leahy, 2007). The creation of a reliable and validated measure of safeness within the therapeutic relationship allows research to explore these suggested mechanisms empirically.

Next steps from this research could include replicating the results in this study with a more diverse clinical sample and completing qualitative research on the views of both client and therapist towards the FSTRQ. To confirm the validity and reliability of the FSTRQ, we would need to replicate our results using a wider range of participants, including participants from secondary care units and inpatient services, and assess safeness ‘live’ during courses of ongoing treatment, rather than retrospectively. Further studies should robustly explore whether safeness can be usefully and statistically considered as a separate construct or whether this is merely a different way of describing the therapeutic alliance (final measure available for this use: File S2 Supplementary File).

The FSTRQ could be useful in clinical settings as a measure of how safe someone feels in their relationship with their therapist. The features associated with FSTRQ delivery, such as timing of administering the FSTRQ and how many repetitions to complete within a cycle of therapy, have not been confirmed within this study. However, reasonable test–retest reliability and the transdiagnostic nature of this measure suggest that it could feasibly be used as a continuous measure within a period of therapy to help the therapist ascertain how safe their client feels in the therapeutic relationship, or to evaluate the quality of a relationship at the end of treatment. Use within ongoing therapy would need careful framing to minimise the impact of demand characteristics on the provision of honest feedback; however, this use during therapy would be consistent with existing use of alliance rating scales, with shared in‐session ratings using the Session Rating Scale showing no significant differences to unshared feedback ratings (Reese et al., 2013). This information could inform clinical practice, highlighting a potential target for intervention and/or situations where therapists should adapt their stance or behaviour to support increased safeness. Further research and guidance such as advice on how to improve safeness in the therapeutic relationship would need to be incorporated to increase its utility as a tool to inform clinical practice.

## CONCLUSIONS

The FSTRQ is a reliable and valid measure to assess feelings of felt safeness in a client's relationship with their therapist. The measure has potential to facilitate research into safeness as a potential mechanism for therapeutic change and for use as a tool to inform clinical practice. The utility of the measure would be improved by further research to learn from patient/clinician feedback on the measure, consider reliability and validity with other groups and specific clinical populations, and explore validity of the construct as a separate facet of the therapeutic relationship.

## AUTHOR CONTRIBUTIONS

**Emily Hesselink:** Conceptualization; methodology; investigation; formal analysis; data curation; project administration; visualization; writing – original draft; writing – review and editing. **Jasmin Langdon‐Daly:** Conceptualization; methodology; writing – review and editing; resources; supervision.

## CONFLICT OF INTEREST STATEMENT

The authors declare no conflicts of interest.

## ETHICS STATEMENT

Ethics approval for this study was gained from the University of Bath Social Sciences Research Ethics Committee. Reference: 8536.

## Supporting information


**Figure S1:** Flow diagram of excluded data.

**File S2:** Felt Safeness in Therapeutic Relationship Scale.

## Data Availability

The data that support the findings of this study are openly available in the Open Science Framework under the main project at https://doi.org/10.17605/OSF.IO/YEQ74.
