# Supplementary material for: Development and validation of the felt safeness in the therapeutic relationship questionnaire
Source: Psychol Psychother. 2026 May 13;99(4):1292–308. doi: 10.1111/papt.70078 (PMC13749245; doi:10.1111/papt.70078)
Supplement: Supplementary file 1 [file PAPT-99-1292-s001.docx]

***Supplementary Figure 1:*** *Flow diagram of excluded data*

Participants recruited through General methods (eg. Social media)

(n = 135)

Participants recruited through Prolific

(n = 135)

Total, reliable participant responses

(n = 226)

Reliable responses

(n = 97)

Factor analysis and correlation

(n = 220)

Test-retest dataset

(n = 128)

Reliable responses

(n = 129)

Excluded:

Fraudulent activity indicators (n=6)

Excluded:

Fraudulent activity indicators (n=8)

Failing attention checks (n=6)	Incomplete answers (n=24)

Excluded due to 2^nd^ instance incomplete (n=92)

Excluded due to missing answers (n=6)
