# Supplementary material for: Development and validation of the felt safeness in the therapeutic relationship questionnaire
Source: Psychol Psychother. 2026 May 13;99(4):1292–308. doi: 10.1111/papt.70078 (PMC13749245; doi:10.1111/papt.70078)
Supplement: Supplementary file 2 [file PAPT-99-1292-s002.docx]

Felt Safeness in the Therapeutic Relationship Scale

We are interested in how people experience positive feelings and emotions in their relationship with their therapist. Below are a series of statements about how you may feel in your relationship with your therapist

Please read each statement carefully and choose the number that best describes how you feel.

|  | Almost never | Rarely | Sometimes | A lot of the time | Almost all the time |
| --- | --- | --- | --- | --- | --- |
| I feel content with my therapist |  |  |  |  |  |
| I feel easily soothed by my therapist |  |  |  |  |  |
| I feel connected to my therapist |  |  |  |  |  |
| I feel that my therapist cares about me |  |  |  |  |  |
| I feel secure in my relationship with my therapist |  |  |  |  |  |
| I feel valued with my therapist |  |  |  |  |  |
| I feel a sense of belonging when I am with my therapist |  |  |  |  |  |
| I feel accepted by my therapist |  |  |  |  |  |
| I feel understood by my therapist |  |  |  |  |  |
| I feel a sense of warmth in my relationship with my therapist |  |  |  |  |  |
| I find it easy to feel calmed by my therapist |  |  |  |  |  |

SCORING Simply add the items

| 1 | 2 | 3 | 4 | 5 |
| --- | --- | --- | --- | --- |
| Almost never | Rarely | Sometimes | A lot of the time | Almost all the time |

Maximum score= 55

Reference

Hesselink, E. & Langdon-Daly, J. (2026) Development and validation of the felt safeness in the therapeutic relationship questionnaire. *Psychology and Psychotherapy: Theory, Research and Practice.* doi: 10.1111/papt.70078
